# Cholera outbreak amidst urban flooding in Karachi: An emergency condition

**DOI:** 10.1016/j.amsu.2022.104365

**Published:** 2022-08-18

**Authors:** Shafaq Taseen, Sara Jawed, Aqleema Mohammad Ameen

**Affiliations:** Karachi Medical and Dental College, Block M North Nazimabad Town, Karachi, Karachi City, Sindh, 74700, Pakistan

Urban Flooding stems from the torrential spells of rainfall overwhelming the capacity of drainage systems in an area of dense population leading to global concerns for economic and social damages. Recent meteorological conditions in Pakistan have resulted in surplus rainfall across the Sindh province inundating it most populous and financial capital, Karachi due to downpour [1]. The Provincial Disaster Management Authority PDMA reported 26 deaths from June 20 to 10 July [1]. With devastating damages to both public and private infrastructure and property, urban flooding has also evoked serious concerns for health and sanitation in the metropolitan setup. Direct contact with drainage and rainwater augmented the threats to vector-borne diseases such as malaria, dengue, Hepatitis A, leptospirosis, and water-borne such as typhoid, diarrhea, cholera, and other gastrointestinal infections, particularly in a low - middle-income country with poor hygiene and sanitation control [2].

After the havoc burdened by the COVID-19 pandemic exposing the vulnerability of the healthcare system worldwide, a new outbreak of Cholera was reported by WHO in Sindh, Karachi after 234 confirmed cases from 15 January to May 27, 2022 [3]. Cholera is an acute infection caused by the bacterium Vibrio Cholera upon contact with contaminated food or water. It is associated with episodes of watery diarrhea and is virulent with high rates of mortality if untreated [2,3]. Climate changes such as heavy rain, followed by floods and damage to sanitary infrastructure accelerates cholera surge due to expediting contact of contaminated water with human activities [4]. Thus, the current situation of urban flooding in Karachi favors the occurrence, growth, and transmission of Cholera.

About 70% public and household water sources of Karachi was found be contaminated with Cholera including *E. coli* (55%), Coliform (90%), and Shigella on testing, making 91% of the water supply unsafe for drinking [5].

Unsuitable sanitary and hygiene (WASH) practices, improper waste disposal, close-quarter residence in more than 600 slums [6], and millions of people with no awareness, resources, or even motivation have been predisposed to the risk of an intensified outbreak. Together with casualties and the spread of vector and water-borne diseases, flood water has imposed a threat to lives and is quite alarming as similar patterns of infection including diarrheal diseases and dengue were reported in 2020 after record-breaking rainfall in Karachi as a result of inadequate sewage treatments and contaminated water supply [7]. Considering an already burdened economy and weakened health setting in low-middle income countries any neglect of preventive measures and efficient control strategies may cause a sharp rise in disease progression jeopardizing millions of individuals at stake. The cases reported so far suggest a possible outbreak, hence, strategies to efficiently counter Cholera must be devised. The combination of prevention and adequate measure for control can refrain from the increasing risk of widespread infections including Cholera and could limit the existing burden of floodwater calamities to a reasonable extent. Thus, to combat the current alarming condition in Sindh and Karachi, this article made some recommendations:

1) Awareness campaigns for management using advertisement and pamphlets emphasizing primary health and hygiene: frequent washing of hands, use of boiled drinking water, thoroughly washing of fruits and vegetables, covering food from flies, ensuring fully cooked home-made food with clean hands and utensils, use of disinfectants to clean floors and distancing from the crowd and avoid using public toilets could be effective to limit the transmission. Advertisements demonstrate steps to boil water or teach household treatment of unclean water with chlorine products (granules and tablets) or household bleach [8]. WASH interventions and health surveillance programs are to be initiated by the government.
2) Discard food that comes in contact with floodwater or rainwater. Also, avoid eating food with unusual odor or color and refrain use of any perishable food that has not been frozen or adequately refrigerated for at least 4 h given the power cutoff during a rainstorm [9]. Avoiding street food that possibly gets contaminated with direct flood water or flies could give positive outcomes in restricting the advancement of the number of cases.
3) Safe Water treatment interventions to be made and monitoring of quality water supply amongst the households and public settings including tertiary clinics and hospitals. Installation of filters and water cleaning plants by the government. Doorstep supplies of sealed mineral water, soaps, hand wash, sanitizers, and disinfectants particularly in slum areas to increase access to basic hygiene kits.
4) Refraining direct contact with floodwater and dispose of any clothing that comes in direct contact with it to minimize the possibility of flu, fever, skin rash, cholera, dengue, and other infectious diseases.
5) Diarrheal death are much more prevalent among infants younger than 5 years [10] thus, maternal guidance should be provided to protect infants from cholera through regular baths, hand washing, wipes, clean drinking water, boil bottles, and appropriate feeding. For already infected children who acquire diarrhea immediate ORS and ample water intake to be regulated to prevent severe dehydration. Material support should provide by government to ensure the availability of supplies including ORS, zinc, selected antibiotics for case management.
6) Earlier diagnosis, antibiotics, vaccination, oral hydrating solution therapy, and online consultations to be provided to the affected individuals. Oral Cholera Vaccine (OCV) administration [3] that significantly reduces the incidence of Cholera [11] and large-scale disinfectant programs are to be introduced.
7) Hospital staff to be alerted and trained to combat the surge of cases. Guidelines should be laid and resources to be accumulated to counter the aggravating situation. The government must also take steps to provide safety kits to front-line staff and workers indulged in restraining efforts against natural calamities. Crisis and case management training and treatment outlines to be deduced in Tertiary Care Centers and hospitals to address the alarming situation of Cholera and other diseases whose epidemic risks are amplified by flooding [5].
8) Crisis management team to be formed and trained to counter the casualties and disasters of floods and spells of the thunderstorm. Special measures were taken to access the situation and launch a volunteer force to enable access to help and hospital facility in case of emergencies. Rescue teams to be mobilized to operate in areas submerged in flood water. Government to accommodate inhabitants with shelter, food supply, and safe water.

## Ethical approval

N/A.

## Sources of funding

None.

## Author contribution

Shafaq Taseen conceived the idea, Shafaq Taseen and Sara Jawed retrieved the data and did write up of an article; and Aqleema Mohammad Ameen did the proof-reading and submission. All authors approve final version of the article.

## Registration of research studies

N/A.

1. Name of the registry: N/A.

2. Unique Identifying number or registration ID: N/A.

3. Hyperlink to your specific registration (must be publicly accessible and will be checked): N/A.

## Guarantor

N/A.

## Consent

N/A.

## Declaration of competing interest

None.
